# Famine Exposure Has Disease‐Specific, Age‐Specific and Sex‐Specific Effects on Different Non‐Communicable Diseases in Later Life: Longitudinal Evidence From China

**DOI:** 10.1111/mcn.70276

**Published:** 2026-10-27

**Authors:** Mengling Cheng, Nicolas Sommet, Marko Kerac, Daniela S. Jopp, Dario Spini

**Affiliations:** ^1^ School of Social and Public Administration East China University of Science and Technology Shanghai China; ^2^ Swiss Centre of Expertise in Life Course Research University of Lausanne Lausanne Vaud Switzerland; ^3^ Centre for Maternal Adolescent Reproductive & Child Health (MARCH), Department of Population Health London School of Hygiene & Tropical Medicine London UK; ^4^ Institute of Psychology; Research Centre for Psychology of Health, Aging and Sport Examination; Swiss Centre of Expertise in Life Course Research University of Lausanne Lausanne Vaud Switzerland

**Keywords:** China, famine exposure, life course, longitudinal, non‐communicable diseases, older age

## Abstract

Malnutrition is a major global challenge with both short‐ and long‐term health impacts. Studies of historical famines have provided evidence of an association between famine exposure and risk of non‐communicable diseases (NCDs) in later life. However, whether famine exposure has heterogeneous effects on different later‐life NCDs remains unclear. In this study, we investigated whether the effects of exposure to the Chinese Famine of 1959–1961 on later‐life NCDs were disease‐specific, age‐specific, and sex‐specific. We conducted a secondary analysis of data from a large‐scale, nationally representative panel study, i.e., the China Health and Retirement Longitudinal Study (CHARLS, 2011–2020, *N* = 11,047 adults aged 45 years and older). We measured famine exposure using self‐reported famine experience, life stage using age at famine exposure, and NCDs using 14 chronic conditions. Three main findings emerged. First, the effects of famine exposure for developing NCDs in later life were disease‐specific, with 20%–29% higher odds of digestive disease, memory‐related disease, arthritis, kidney disease and dyslipidemia, versus 10% higher odds of heart disease and diabetes. Second, the effects of famine exposure were age‐specific, with exposure in utero or at ages 0–2 years having the largest impacts on 8 of the 14 later‐life conditions (diabetes, stroke, lung disease, liver disease, digestive disease, cancer, kidney disease, and arthritis), whereas exposure at ages 3–5 years having the largest impact on psychiatric disease and memory‐related disease in later life. Conversely, famine exposure during adulthood was associated with lower odds of various NCDs. This divergence reflects a balance of competing forces: for individuals exposed prior to adulthood, severe developmental damage overshadows the healthy survivor effect, increasing later‐life NCD risk; for individuals exposed during adulthood, completed physiological development minimized programming damage, allowing the healthy survivor effect to dominate. Third, the famine effects were sex‐specific for later‐life cardiometabolic diseases (dyslipidemia, heart disease and stroke), with famine exposure having larger impacts on women than men, pointing to the survivor advantage among men. Future studies should investigate underlying biological mechanisms, intergenerational pathways of transmission, and long‐term growth and body composition in survivors.

## Key Messages

- Famine exposure has disease‐specific effects on non‐communicable diseases in later life.
- Famine exposure in utero or at ages 0–2 years most affects physical health, while exposure at ages 3–5 years most impacts mental health in later life.
- Women face a higher risk than men for famine‐related cardiometabolic diseases.

## 1 Introduction

Malnutrition and food insecurity remain pressing global challenges, exacerbated by climate change, natural disasters, wars, and pandemics (World Food Programme 2021; World Health Organization 2024). However, at the most extreme and devastating end of this nutritional spectrum is famine, defined as an event identifiable by excess mortality caused by starvation (Bhatia 1967; Rubin 2016; Sen 1981). While the short‐term effects of such extreme nutritional deprivation are severe and visible, there is also increasing awareness of its long‐term impacts. Specifically, famine exposure has been increasingly recognized as a profound risk factor for non‐communicable diseases (NCDs) in later life (Gray et al. 2021; Kiosia et al. 2024; Song et al. 2025).

Previous famine studies across diverse historical contexts and locations, such as the Dutch Hunger Winter and the Chinese Famine, have consistently demonstrated that famine exposure significantly increased risk of NCDs in later life. For example, fetal famine exposure has been linked to an elevated risk of later‐life cardiometabolic diseases and fatty liver in Chinese cohorts (Xu et al. 2022; Zhang et al. 2022), as well as an increased risk of later‐life anxiety and depression in Dutch cohorts (Hilberdink et al. 2023). This body of work provides three valuable insights into the link between famine exposure and later‐life NCDs. First, exposure to famine early in life can significantly influence health trajectories, contributing to a higher risk of NCDs in later life (Lumey et al. 2024). Second, exposure during critical developmental periods, sometimes even quite chronologically tight periods, can affect later life NCD outcomes: for example, exposure during the first two trimesters of gestation versus the final trimester (Ramirez and Haas 2022). Third, famine effects might be more pronounced among older women than men (Wang et al. 2016).

Consistent with the aforementioned studies, previous work found that exposure to the Chinese Famine of 1959–1961 was associated with a higher risk of later‐life multimorbidity, defined as the count of chronic diseases within an individual (Cheng et al. 2023). However, combining NCDs may obscure the distinct, disease‐specific biological mechanisms triggered by famine exposure. For instance, famine conditions constrain homeostatic capacity, which aids short‐term survival but maladapts the body to later‐life food abundance, increasing the metabolic load and risk of cardiometabolic diseases over time (Wells 2018). Protein‐calorie deficits impair renal blood flow and glomerular filtration rates, laying the direct physiological groundwork for subsequent chronic kidney disease (Liu et al. 2024). Furthermore, it is likely that these disease‐specific impacts can vary, depending on the timing of famine exposure. However, few studies have examined famine exposure beyond fetal and childhood, leaving a critical gap regarding how individuals exposed to famine in adolescence or adulthood were affected (Wang et al. 2016; Yu et al. 2017). Moreover, given that women and men have differing risks of specific NCDs, the associations between famine exposure and NCDs can also vary to some extent. Therefore, it remains unclear whether and how famine exposure has heterogeneous effects across later‐life NCDs, age and sex. It is important to disentangle the heterogeneity to better understand the mechanisms underlying famine exposure and specific chronic conditions among older adults (Gray et al. 2021).

In the current study, we built on our previous research and investigated whether famine exposure has heterogeneous effects on specific NCDs in later life. We analyzed data from a large‐scale, nationally representative, panel study, i.e., the China Health and Retirement Longitudinal Study (CHARLS, 2011–2020). Our objectives were to investigate whether the effects of exposure to the Chinese Famine of 1959–1961 on later‐life NCDs were disease‐, age‐, and sex‐specific.

## 2 Methods

### 2.1 Study Setting

This study examined the 1959–1961 Chinese Famine, a crisis often considered one of the largest famines in human history. The unprecedented scale of this crisis stemmed from a confluence of factors: its prolonged 3‐year duration, nationwide geographic extent, and the immense magnitude of the affected population (St Clair 2005). Furthermore, due to the historical household registration (hukou) system and urban‐biased food rationing policies during that era, rural areas experienced substantially more severe food shortages (Lin and Yang 2000). During the famine, grain production plummeted by approximately 25%, resulting in nearly 30 million excess deaths (Ashton et al. 1992). Total grain production and per capita output did not recover to pre‐famine levels until 1965 and post‐1970, respectively (Smil 1999), reflecting a prolonged and gradual return to adequate nutritional intake.

### 2.2 Data and Participants

The data source for this study was the China Health and Retirement Longitudinal Study (CHARLS). Employing a stratified, multistage probability sampling strategy to ensure national representativeness (Zhao et al. 2014), CHARLS has collected biennial data since 2011 on a cohort of approximately 26,000 individuals aged 45 years and older in China. We constructed our dataset by combining the 2014 Life History survey with all five core CHARLS waves (2011, 2013, 2015, 2018, 2020).

Eligible participants were those who: (1) were born between January 1919 and September 1960 (ensuring potential exposure to the famine period from in utero to age 40), and (2) provided complete information for key study variables, including NCD status, sociodemographics, famine experience, and financial and health histories in childhood and adulthood. The final sample comprised 38,553 observations from 11,047 older adults (for the flow diagram of study participants, see Figure 1).

**Figure 1:**
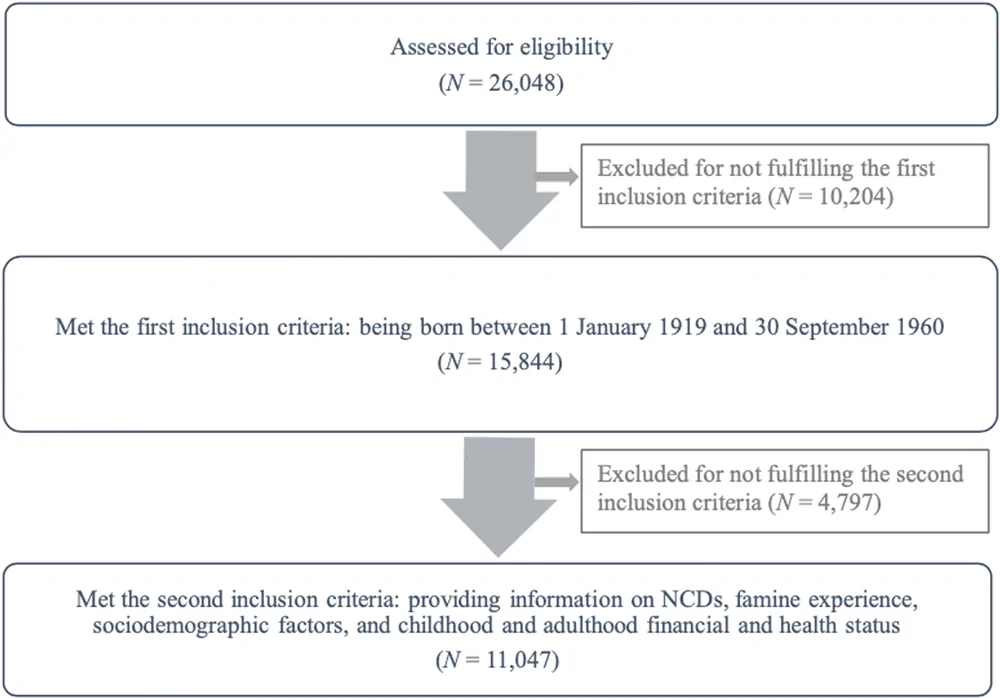
Flow diagram of the final sample. The flowchart details the stepwise inclusion and exclusion criteria used to derive the final analytic sample. From an initial pool of 26,048 participants assessed for eligibility in the China Health and Retirement Longitudinal Study (CHARLS), 10,204 were excluded for not meeting the primary birth cohort criteria (born between January 1, 1919, and September 30, 1960). Of the remaining 15,844 participants, a further 4797 were excluded due to missing information regarding non‐communicable diseases (NCDs), famine experience, sociodemographic factors, and childhood and adulthood financial and health status. The final analytic sample comprised 11,047 participants.

### 2.3 Measurement

*Non‐communicable diseases*. Participants reported whether a physician had ever diagnosed them with any of 14 chronic conditions, including hypertension, dyslipidemia, diabetes, heart disease, stroke, lung disease, asthma, liver disease, digestive disease, psychiatric disease, memory‐related disease (dementia and brain atrophy), cancer, kidney disease, and arthritis. If participants are deemed cognitively unable to answer these diagnosis questions accurately, a knowledgeable proxy (e.g., spouse or adult child) answers the questions on their behalf. Responses were coded dichotomously (0 = *no*; 1 = *yes*).

*Famine exposure*. In line with the operationalization used in previous studies (e.g., Cheng et al. 2023), we created a dichotomous variable to classify participants based on their famine exposure status. In the CHARLS survey, exposure is determined by asking participants whether they experienced starvation during the 1959–1961 period. For individuals who were in utero or in early childhood during the famine years, this self‐report likely reflects family‐level exposure as remembered or transmitted through family oral history. To ensure consistency and minimize interviewer‐induced variation, the CHARLS survey was administered face‐to‐face by rigorously trained interviewers utilizing standardized computer‐assisted personal interview (CAPI). Individuals who reported famine exposure between 1959 and 1961 were categorized as exposed, while those who did not were categorized as unexposed (0 = *no*; 1 = *yes*).

*Life stage*. To rigorously control for age differences introduced by the 3‐year duration of the famine, we anchored our analysis to a single reference date: the famine's onset (January 1, 1959). We calculated each participant's age at this onset date to categorize them into seven life‐stage cohorts: in‐utero, first 1000 days (0–2 years), pre‐school (3–5 years), primary school (6–9 years), adolescence (10–18 years), young adulthood (19–23 years), and adulthood (24–40 years). Including individuals exposed during adolescence and adulthood serves two purposes. First, life course epidemiology suggests that the timing of an exposure is critical and that the effect of an exposure during a specific period may differ from the effect of the same exposure during another period (Ben‐Shlomo 2002). Second, the effects of famine exposure beyond fetal period and early childhood remain underexplored (Wang et al. 2016; Yu et al. 2017). The cutoffs for defining and distinguishing these cohorts are adapted from life course frameworks commonly used in major global policies, which emphasize distinct developmental windows, and the categorization strategy has been validated in previous work (Cheng et al. 2023).

*Famine exposure–life stage*. We used a combined variable based on famine exposure and developmental life stage to create eight groups. These included an unexposed reference group and seven groups exposed to famine in utero, during the first 1000 days of life (0–2 years), in pre‐school (3–5 years), in primary school (6–9 years), in adolescence (10–18 years), in young adulthood (19–23 years), or in adulthood (24–40 years). We did not stratify or weight the sample to force equal numbers across these categories; rather, the sample sizes reflect the demographic distribution of the CHARLS cohort. Despite this natural variation in group sizes, all categories provided sufficient observations for statistical analysis.

*Covariates*. To improve model precision, we incorporated covariates including age, sex (−0.5 = *man*, +0.5 = *woman*), later‐life residence (0 = *rural*, 1 = *urban*), current marital status (0 = *not married*, 1 = *married*), working status (0 = *not working*, 1 = *working*), family financial status in childhood (from 1 = *much better* to 5 = *much worse than families in the same community*), educational attainment (1 = *less than upper secondary*, 2 = *upper secondary and vocational*, 3 = *tertiary*), household income (from 1 = *bottom 10%* to 10 = *top 10%*), number of diseases in childhood, and number of diseases in adulthood.

### 2.4 Analytical Strategy

We structured our panel data in a person‐period long format and employed growth curve modeling. In this modeling framework, the survey round (captured by the wave variable, ranging from 1 to 5) serves as the fundamental metric of time. Because growth curve models inherently accommodate unbalanced data structures, this approach corrects for the influence of different years of enrollment and missing waves, ensuring that the timing of the survey is fully integrated into the estimation. Specifically, in our models, 38,553 wave‐specific observations (level 1) were nested within 11,047 participants (level 2).

We conducted a series of two‐level growth curve models. First, we regressed the outcome variable of each NCD on the focal predictor of famine exposure to assess the heterogeneity in the associations between famine exposure and later‐life NCDs across disease domains. Second, we regressed the outcome variable of each NCD on the focal predictor of famine exposure‐life stage to investigate the heterogeneity in the associations between famine exposure and later‐life NCDs across life stage at exposure. Third, we regressed the outcome variable of each NCD on the focal predictors of famine exposure, sex, and the interaction between famine exposure and sex to examine the heterogeneity in the associations between famine exposure and later‐life NCDs by sex. We compared the log odds ratios based on average predictions among women and men, i.e., ln(odds(1)/odds(0)). We further stratified the results by sex if the comparisons were statistically significant.

All models were fitted using R (v4.3.3). The corresponding analysis code is publicly available on the Open Science Framework (OSF), https://osf.io/4uazs/?view_only=0d66d022e66147cf88f7d49584466630.

### 2.5 Ethics Statement

This research was conducted in accordance with the ethical principles of the Declaration of Helsinki. The study protocol received ethical approval from the London School of Hygiene & Tropical Medicine Ethics Committee (Reference Number 28001). The analysis utilized data from the China Health and Retirement Longitudinal Study (CHARLS), which is fully open access and available to researchers to download from the CHARLS website.

## 3 Results

The analytical sample comprised 38,553 observations from 11,047 older adults. A total of 83% of the participants (*N* = 9221) were exposed to famine, while 17% of the participants (*N* = 1826) were unexposed during famine years. Table 1 details the number of participants stratified by sex and famine exposure at each life stage. Table 2 details the sample characteristics stratified by famine exposure status.

**Table 1:** Number of study participants, stratified by sex, life stage during the 1959–1961 Chinese Famine, and famine exposure status.

	Overall (*N* = 11,047)		Male (*N* = 5,427)		Female (*N* = 5,620)	
	Exposed (*N* = 9221)	Unexposed (*N* = 1826)	Exposed (*N* = 4610)	Unexposed (*N* = 817)	Exposed (*N* = 4611)	Unexposed (*N* = 1009)
In‐utero	311	81	144	37	167	44
The “first 1000 days” (0–2 years)	910	225	447	102	463	123
Pre‐school (3–5 years)	1533	318	768	124	765	194
Primary school (6–9 years)	2042	286	978	122	1064	164
Adolescence (10‐18 years)	2988	486	1,546	216	1442	270
Young adulthood (19–23 years)	807	238	434	116	373	122
Adulthood (24–40 years)	630	192	293	100	337	92

*Note: N* represents the number of participants.

Categorizations of life stage are based on the participant's age at the onset of famine (January 1, 1959).

Individuals who reported famine exposure between 1959 and 1961 were categorized as exposed, while those who did not were categorized as unexposed.

**Table 2:** Sample characteristics, stratified by exposure to the 1959–1961 Chinese famine.

	Overall (*N* = 11,047)	Exposed (*N* = 9221)	Unexposed (*N* = 1826)
Females (%)	50.9	50.0	55.3
Age (mean; in years)	65.8 (SD = 7.7)	65.7 (SD = 7.5)	66.6 (SD = 8.6)
Childhood family financial status(mean; 1 = *much better*, 5 = *much worse than other families*)	3.6 (SD = 0.9)	3.6 (SD = 0.9)	3.2 (SD = 0.9)
Equivalized Income (mean; in CNY)	13,717 (SD = 26, 252)	13,169 (SD = 26,678)	16,484 (SD = 23,796)
Educational levels (%)			
Less than lower secondary	91.0	91.4	88.7
Upper secondary or vocational	7.7	7.5	9.0
Tertiary	1.3	1.1	2.4
Number of childhood health conditions (mean)	0.1 (SD = 0.3)	0.1 (SD = 0.3)	0.1 (SD = 0.3)
Number of adulthood health conditions (mean)	0.4 (SD = 0.7)	0.4 (SD = 0.7)	0.4 (SD = 0.7)
Rural residence in later life (%)	62.3	63.2	57.9
Currently married (%)	84.7	85.1	82.3
Currently working (%)	57.5	58.8	51.4
Participants diagnosed with specific non‐communicable diseases (%)			
Hypertension	30.4	30.0	32.1
Dyslipidemia	11.8	12.0	10.5
Diabetes	7.8	7.7	7.9
Heart disease	14.6	14.7	14.2
Stroke	3.4	3.4	3.5
Lung disease	11.9	12.0	11.3
Asthma	5.3	5.4	5.0
Liver disease	3.9	4.1	3.2
Digestive disease	22.5	23.4	18.1
Psychiatric disease	1.7	1.6	1.8
Memory‐related disease	2.4	2.5	2.0
Cancer	0.9	0.9	1.0
Kidney disease	6.5	6.7	5.6
Arthritis	36.1	37.2	30.4

*Note:* Data are presented as percentages (%) for categorical variables and as means with standard deviations (SD) for continuous variables.

Individuals who reported famine exposure between 1959 and 1961 were categorized as exposed, while those who did not were categorized as unexposed.

CNY refers to Chinese Yuan.

Equivalized income was calculated by adjusting total household income by household size using the square root scale.

### 3.1 Disease‐Specific Associations Between Famine Exposure and Later‐Life NCDs

In our models adjusted for covariates, we observed disease‐specific associations between famine exposure and later‐life NCDs. Specifically, compared with unexposed individuals, exposed individuals had a 20%–29% higher odds of digestive disease, memory‐related disease, arthritis, kidney disease, and dyslipidemia (adjusted ORs ranged from 1.20, 95% CI [1.10, 1.30] to 1.29, 95% CI [1.21, 1.37]), and a 10% higher odds of heart disease and diabetes (adjusted ORs of 1.10, 95% CI [1.02, 1.18] and 1.10, 95% CI [1.01, 1.19]), respectively. The results were not statistically significant for liver disease, lung disease, asthma, cancer, stroke, psychiatric disease, and hypertension. Full results are reported in Figure 2.

**Figure 2:**
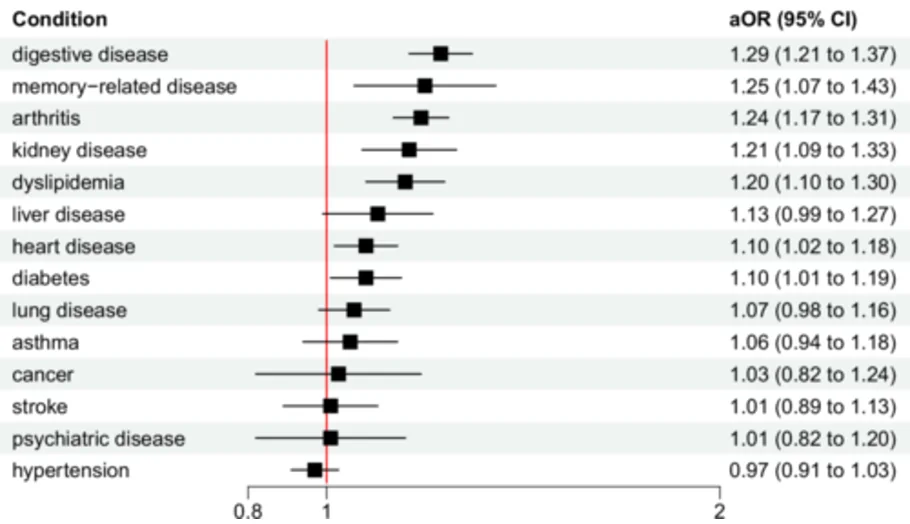
Disease‐specific associations between famine exposure (at any age) and the risk of 14 later‐life non‐communicable diseases. Black squares denote the point estimates for the adjusted Odds Ratios (aOR), and the horizontal lines represent the corresponding 95% Confidence Intervals (CI). The vertical red line indicates the null value (aOR = 1.00). Estimates positioned to the right of the red line signify an increased risk associated with the exposure. Confidence intervals that do not intersect the null line indicate statistically significant associations at the *p* < 0.05 level. Models were adjusted for age, sex, later‐life residence, current marital status, working status, family financial status in childhood, educational attainment, household income, number of diseases in childhood, and number of diseases in adulthood.

### 3.2 Age‐Specific Associations Between Life Stage at Famine Exposure and Later‐Life NCDs

In our models using the famine exposure‐life stage as the predictor and adjusting for covariates, we observed age‐specific associations between life stage at famine exposure and later‐life NCDs, *χ* ^2^ (7, *N* = 38,553) > 58.00, *p* < 0.001 (Figure 3). First, compared with unexposed individuals, those exposed in utero or at ages 0–2 years had the highest odds for 8 of the 14 later‐life conditions studied: diabetes, stroke, lung disease, liver disease, digestive disease, cancer, kidney disease, and arthritis (adjusted ORs ranged from 1.43, 95% CI [1.28, 1.58] to 2.49, 95% CI [1.38, 3.60]). Second, compared with unexposed individuals, those exposed at ages 3–5 years had the highest odds for developing psychiatric disease (adjusted OR = 1.43, 95% CI [1.09, 1.77]) and memory‐related disease (OR = 2.02, 95% CI [1.53, 2.51]) in later life. Third, compared with unexposed individuals, those exposed before age 10 had consistently higher odds for dyslipidemia (adjusted ORs ranged from 1.60, 95% CI [1.31, 1.89] to 1.64, 95% CI [1.43, 1.85]) and heart disease (adjusted ORs ranged from 1.26, 95% CI [1.13, 1.39] to 1.39, 95% CI [1.26, 1.53]) in later life. Fourth, famine exposure at a later life stage was associated with lower odds of various conditions in later life. Individuals exposed after age 18 had lower odds for metabolic and cardiovascular disease in later life—including hypertension, dyslipidemia, diabetes, stroke, and heart disease—as well as lung disease, liver disease, psychiatric disease, cancer and kidney disease (adjusted ORs ranged from 0.31, 95% CI [0.19, 0.43] to 0.80, 95% CI [0.67, 0.93]). Those exposed after age 23 had lower odds for asthma, digestive disease, memory‐related disease and arthritis in later life (adjusted ORs ranged from 0.56, 95% CI [0.44, 0.68] to 0.80, 95% CI [0.70, 0.91]).

**Figure 3:**
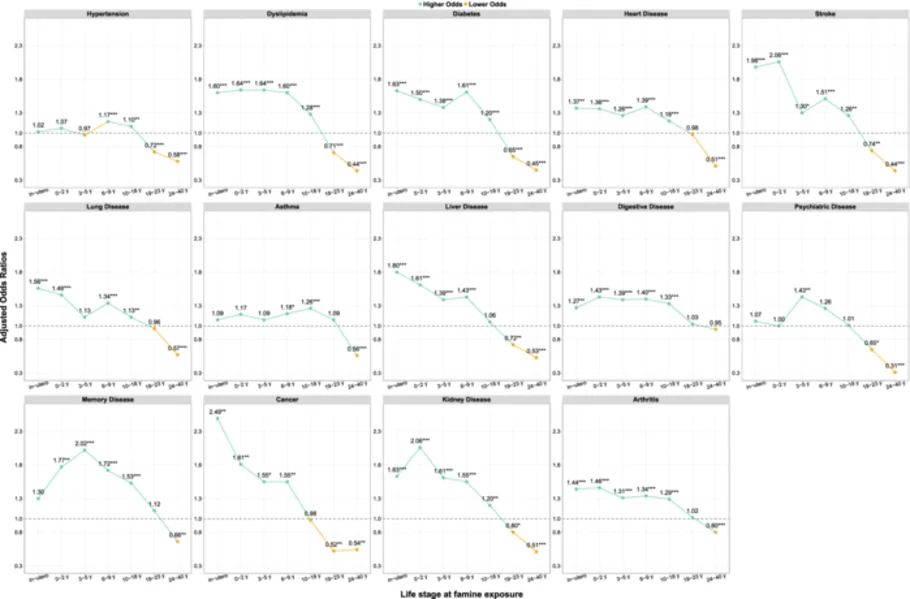
Age‐specific associations between life stage at famine exposure and the risk of 14 later‐life non‐communicable diseases. The x‐axis categorizes the specific life stage during which participants were exposed to the famine (ranging from in utero to 24–40 years of age). The y‐axis displays the adjusted Odds Ratios (aOR) for each chronic disease. The horizontal dashed gray line denotes the null value (aOR = 1.0). Estimates positioned above the dashed gray line (i.e., green data points) signify higher odds of disease development (aOR > 1.0), whereas estimates positioned below the dashed gray line (i.e., orange data points) signify lower odds (aOR < 1.0) relative to the unexposed cohort. Confidence intervals that do not intersect the null line indicate statistically significant associations at the *p* < 0.05 level. Asterisks indicate the level of statistical significance: *p < 0.05, **p < 0.01, and ***p < 0.001. Models were adjusted for age, sex, later‐life residence, current marital status, working status, family financial status in childhood, educational attainment, household income, number of diseases in childhood, and number of diseases in adulthood.

### 3.3 Sex‐Specific Associations Between Famine Exposure and Later‐Life NCDs

We found a gendered pattern of famine effect on later‐life cardiometabolic diseases including dyslipidemia, heart disease and stroke (ln(odds(1)/odds(0)) ranged from 0.12, 95% CI [0.05, 0.19] to 0.23, 95% CI [0.16, 0.31]. We further stratified the results by sex. Famine effects on dyslipidemia, heart disease and stroke in later life tended to be larger among women than men (Figure 4).

**Figure 4:**
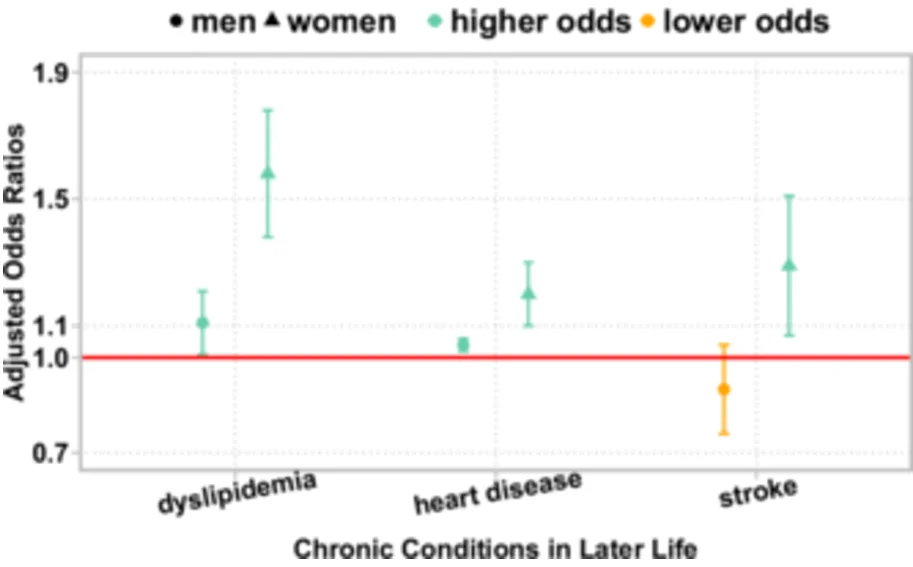
Sex‐specific associations between famine exposure and the risk of specific later‐life non‐communicable diseases. The plot displays the adjusted Odds Ratios (aOR) for dyslipidemia, heart disease, and stroke, stratified by sex. Circles represent point estimates for men, while triangles represent point estimates for women. The vertical error bars denote the corresponding 95% Confidence Intervals (CI). The horizontal red line indicates the null value (aOR = 1.0). Estimates positioned above the red line (i.e., green data points) signify higher odds of disease development (aOR > 1.0), whereas estimates positioned below the red line (i.e., orange data point) signify lower odds (aOR < 1.0). Models were adjusted for age, later‐life residence, current marital status, working status, family financial status in childhood, educational attainment, household income, number of diseases in childhood, and number of diseases in adulthood.

### 3.4 Sensitivity Analyses

We conducted several sets of sensitivity analyses to assess the robustness of our findings. First, we compared the mean age of onset for the 14 chronic diseases between the exposed and unexposed groups. The exposed group exhibited a significantly earlier age of onset for six specific morbidities (Table S1): psychiatric disease (−4.95 years), asthma (−3.93 years), kidney disease (−2.27 years), memory‐related disease (−2.18 years), heart disease (−1.15 years), and arthritis (−0.99 years).

Second, we used a combination approach to measure famine exposure. Specifically, we combined subjective self‐reports with an objective measure: provincial‐level excess death rates during the famine years (Lin and Yang 2000). Participants were classified as exposed only if they self‐reported exposure and their residence during famine years matched one of the 28 provinces with documented excess mortality. The results using this combination approach remained consistent with our main findings (Figure S1).

Third, we further adjusted for lifestyle variables (smoking, drinking and physical exercise) and related socio‐economic variables (occupation in addition to education and income). Overall, our primary findings remained consistent (Figure S2). Multicollinearity diagnostics indicated no cause for concern, with all degrees‐of‐freedom‐adjusted generalized variance inflation factors (adjusted GVIF; Fox and Monette 1992) below 2.24 (equivalent to VIF of 5) except for age (2.38–2.60, below the conventional VIF threshold of 10) (Table S2).

Fourth, to address the relatively broad 16‐year span of the adulthood group (24–40 years), we conducted a subgroup analysis. We divided the adulthood period into two narrower bands (24–30 years and 31–40 years). This classification was used because it marks the physiological transition from early adulthood, by which point physiological homeostasis has typically been attained, to established adulthood, during which age‐related physiological dysregulation begins to accumulate (Bogin 2015; Yang and Kozloski 2011). The results support the robustness of our main findings. Both subgroups consistently demonstrated lower odds for the majority of the NCDs compared to unexposed individuals (Figure S3).

Fifth, we restricted to the subgroup of respondents (*N* = 3684) who could clearly indicate their urban (*N* = 561, among whom 393 were exposed) or rural (*N* = 3123, among whom 2646 were exposed) residence during the famine period. The famine effect on diabetes (*p* = 0.038) and arthritis (*p* = 0.040) was significantly stronger in rural environments, which aligns with the historical reality that rural populations suffered greater nutritional deprivation.

Finally, we tested a three‐way interaction (famine exposure × life stage × sex) across all 14 chronic diseases. This three‐way interaction was only statistically significant for heart disease (*p* = 0.009). For women, famine exposure at later life stages, particularly during adolescence and adulthood, was associated with a higher risk of heart disease; for men, exposure during early life conferred the highest risk, whereas exposure during adolescence and adulthood showed a diminishing association (Figure S4). This aligns with findings from the Överkalix cohorts in northern Sweden, which demonstrated that mid‐childhood and adolescence (the prepubertal “slow‐growth” period) represent a highly sensitive window where nutritional exposure drives distinct, sex‐specific cardiovascular and mortality outcomes (Bygren et al. 2014; Pembrey et al. 2014).

## 4 Discussion

Previous studies showed that famine exposure was associated with NCDs in later life; however, the heterogeneity in such association across NCDs domains, age and sex remained unclear. In the current study, we extended previous research by investigating whether famine exposure has heterogeneous effects across different NCDs, age and sex in later life. Our study yielded three main findings, all of which remained robust across multiple sensitivity analyses.

First, we found that the long‐term effects of famine exposure were disease‐specific. Previous studies have linked famine exposure to an increase in the risk of later‐life chronic conditions like heart disease, diabetes, and anxiety (Gray et al. 2021; Lumey et al. 2024). Our findings expand the scope of investigation across 14 distinct later‐life NCDs and demonstrate that famine exposure was associated with higher odds of cardiometabolic, digestive, renal, rheumatologic, and memory‐related conditions in later life.

Second, we found that the effects of famine exposure were age‐specific. Prior research has consistently reported that famine exposure during early life (e.g., in utero, fetal, or early childhood) increases the risk of diseases like cardiovascular disease, diabetes, and cancer (Ramirez and Haas 2022; Taeubert et al. 2024; Zhou et al. 2023). However, few studies have examined famine exposure beyond these stages, leaving a critical gap regarding how individuals exposed to famine in adolescence or adulthood were affected (Wang et al. 2016; Yu et al. 2017). Our study addressed this gap by comparing the effects of famine exposure at different life stages from in utero through adulthood. We found that individuals exposed during early development, particularly in utero and infancy, exhibited higher odds of various later‐life NCDs. Conversely, individuals exposed during adolescence or adulthood exhibited lower odds of various later‐life NCDs.

Third, we found that the effects of famine exposure were sex‐specific for cardiometabolic diseases. Previous studies have documented that famine exposure has more pronounced effects on later‐life obesity, diabetes, and dyslipidemia among women compared to men (Lumey et al. 2009; J. Wang et al. 2016; Y. Wang et al. 2010). In line with this literature, our findings showed that famine effects on later‐life dyslipidemia, heart disease, and stroke tended to be larger for women than for men in the CHARLS cohort.

### 4.1 Underlying Mechanisms

Biological mechanisms help explain the disease‐specific effects of famine exposure. Different physiological systems and organs adapt to famine conditions in distinct ways, including structural impairments, systemic dysregulation, and developmental adaptations (Barker 1995; Hoffman et al. 2017). For instance, famine conditions can cause structural impairments that physically diminish the absorption area of the small intestine (Tappenden 2006) or impair renal blood flow and glomerular filtration rates (Liu et al. 2024), increasing the risk of developing later‐life digestive and chronic kidney diseases, respectively. Furthermore, exposure to famine can also induce systemic dysregulation, such as altering neurotransmitter function linked to adverse memory‐related outcomes (Holden 2007) or triggering gut dysbiosis that initiates autoimmune reactions underlying arthritis (Liu et al. 2021). Additionally, the thrifty phenotype hypothesis posits that famine constrains homeostatic capacity, which aids short‐term survival but maladapts the body to subsequent food abundance, ultimately increasing the metabolic load and risk of cardiometabolic diseases in later life (Wells 2018).

The sensitive period hypothesis helps to explain the increased vulnerability of individuals exposed to famine during specific developmental windows. Environmental shocks during periods of high developmental plasticity become biologically embedded, producing more pronounced health deficits than the same exposures occurring outside these windows (Fawcett and Frankenhuis 2015). During these developmental windows, severe nutritional deprivation can permanently alter organ structure, cellular function, and metabolic pathways, a process referred to as developmental programming (Ben‐Shlomo et al. 2016). Because the developmental phase spanning from in utero to childhood is characterized by rapid growth and organogenesis (Mishra et al. 2010), famine exposure during these stages has profound and often irreversible biological effects. Notably, research has extended these developmental periods into adolescence, as the rapid pubertal maturation of organ systems also occurs during this period (Bateson and Gluckman 2012; Bygren et al. 2014; Pembrey et al. 2014).

We interpret the observed long‐term effects of famine exposure on later‐life NCDs as a dynamic balance between two competing forces: the physiological programming caused by exposure during sensitive periods (which increases disease risk) and the healthy survivor effect (which decreases observed risk). For individuals exposed prior to adulthood, severe developmental damage overshadows the healthy survivor effect, resulting in a net increase in later‐life NCD risk. Conversely, for individuals exposed during adulthood, physiological development was largely complete, minimizing programming damage. Meanwhile, during the Chinese famine, adults frequently prioritized allocating scarce food to infants and young children in the family (Fan et al. 2008), a sacrifice that intensified their own starvation, which could have increased their mortality risk. Furthermore, because the famine occurred in 1959–1961 and CHARLS enrollment spanned 2011–2020, adulthood‐exposed participants were of extreme old age at data collection. Therefore, this group represents an exceptionally robust subset of individuals who survived both the acute starvation of the famine and the natural attrition of aging (Hu et al. 2017). Consequently, the healthy survivor effect becomes dominant, pulling adjusted odds ratios significantly below 1.0.

The heavier later‐life cardiometabolic burden observed in famine‐exposed women is likely driven by intersecting biological, demographic, and cultural mechanisms. Biologically, this disparity is rooted in the frail male phenomenon (Heijmans et al. 2008; Wells 2000). Because males generally mature more slowly than females, they have a wider window of vulnerability to nutritional insults (Thompson 2021). Conversely, females exhibit faster cardiovascular maturation, allowing them to survive but with embedded long‐term cardiometabolic damage (Lorente‐Pozo et al. 2018). This biological divergence drives a profound healthy survivor effect among males (Thurstans et al. 2020, 2022). Highly susceptible to early‐life malnutrition, males experienced selective fetal loss and disproportionate infant mortality during the famine (Song 2012). Consequently, the men who survived the famine represent an exceptionally robust subset of the cohort. This male survival selection was further compounded by a prevailing culture of son preference during the Chinese famine, wherein families preferentially allocated scarce food to sons over daughters (Mu and Zhang 2011). As a result, surviving men benefited from both genetic selection and social buffering, while surviving women endured more severe deprivation. However, because some studies show no long‐term sex differences in mortality (Thurstans et al. 2023), unmeasured socio‐environmental factors may also contribute to the observed sex disparity in risk of later‐life NCDs.

### 4.2 Limitations

We acknowledge several limitations in the current study. First, the assessment of NCDs relied on self‐reported diagnoses rather than direct linkage to medical records (which is unavailable), which might be subject to recall bias and underreporting due to healthcare access disparities. However, the CHARLS protocol mitigates the risk of recall bias by asking knowledgeable proxy respondents (e.g., spouse or adult child) to answer the diagnosis questions on behalf of participants with cognitive impairment. Furthermore, self‐reported NCD data are a standard, widely validated metric in large‐scale, population‐based aging cohorts (Beckett et al. 2000). Second, the retrospective measure of famine exposure might be subject to recall bias and misclassification. However, a recent review indicates that interview‐based assessments of major life events demonstrate acceptable reliability (Monroe and Slavich 2018). Third, despite a robust set of covariates, our models cannot account for factors such as genetic predispositions or birth weight, as data on these factors are unavailable. Fourth, longitudinal data are inherently susceptible to survivor bias. Participants who remain in the cohort are typically healthy survivors, which might attenuate the observed long‐term effects of famine exposure. Fifth, statistically non‐significant findings must be interpreted alongside statistical power. Null findings for rare conditions like cancer may reflect insufficient power, whereas null findings for highly prevalent conditions like hypertension may reflect a true lack of association, although limited precision or small effect sizes remain possible.

### 4.3 Implications for Public Health Research, Policy and Practice

Our study provides six implications for research, public health policy and practice. First, because individual caloric records are unavailable during the Chinese Famine, future studies need to develop granular measures of exposure to quantify nutritional stress. Second, future studies need to explore the biological mechanisms through which famine exposure at different life stages becomes biologically embedded and influences domain‐specific NCDs in later life, while considering the socio‐environmental factors and context (Ciancio et al. 2023). Third, future studies need to investigate possible inter‐generational effects of famine exposure and its underlying mechanisms (Kerac and Cheng 2024). Fourth, future research needs to conduct longer‐term malnutrition and famine follow‐up studies to track survivors, as overt NCDs may not yet be clinically present: measuring long‐term growth and body composition might be one way of identifying early risk (Owino et al. 2019). Fifth, given the highly prevalent conditions (e.g., digestive diseases), even modest relative risks translate to a substantial absolute number of excess clinical cases at the population level, demanding targeted healthcare resources. Sixth, public health policy can leverage the established risk of NCD development as an advocacy tool to strengthen global efforts for preventing and treating hunger.

## 5 Conclusion

Our analysis of a large‐scale, nationally representative panel dataset reveals that the effects of famine exposure were disease‐specific, age‐specific, and sex‐specific, adding important additional specificity information that was lacking in prior research. The findings suggest that future studies are warranted to investigate underlying biological mechanisms, intergeneration pathways of transmission, and long‐term growth and body composition in survivors.

## Author Contributions

MC designed and conceptualized the study. MC conducted data analysis and interpretation, with feedback from NS, MK, DSJ, and DS. MC wrote the first and subsequent drafts of the manuscript, with comments from NS, MK, DSJ, and DS. All authors have read and approved the final manuscript. MC and MK were responsible for the project administration.

## Conflicts of Interest

The authors declare no conflicts of interest.

## Supporting information


**Figure S1:** Disease‐specific associations between famine exposure (at any age) and the risk of 14 later‐life non‐communicable diseases. Participants were classified as exposed only if they self‐reported exposure and their residence during famine years matched to one of the 28 provinces documented to have experienced excess mortality. Black squares denote the point estimates for the adjusted Odds Ratios (aOR), and the horizontal lines represent the corresponding 95% Confidence Intervals (CI). The vertical red line indicates the null value (aOR = 1.00). Estimates positioned to the right of the red line signify an increased risk associated with the exposure. Confidence intervals that do not intersect the null line indicate statistically significant associations at the *p* < 0.05 level. Models were adjusted for age, sex, later‐life residence, current marital status, working status, family financial status in childhood, educational attainment, household income, number of diseases in childhood, and number of diseases in adulthood.
**Figure S2:** Disease‐specific associations between famine exposure (at any age) and the risk of 14 later‐life non‐communicable diseases. Black squares denote the point estimates for the adjusted Odds Ratios (aOR), and the horizontal lines represent the corresponding 95% Confidence Intervals (CI). The vertical red line indicates the null value (aOR = 1.00). Estimates positioned to the right of the red line signify an increased risk associated with the exposure. Confidence intervals that do not intersect the null line indicate statistically significant associations at the p < 0.05 level. Models were adjusted for age, sex, later‐life residence, current marital status, working status, family financial status in childhood, educational attainment, household income, occupation, number of diseases in childhood, number of diseases in adulthood, lifestyle (smoking, drinking, and physical exercise).
**Figure S3:** Age‐specific associations between life stage at famine exposure (eight groups) and the risk of 14 later‐life non‐communicable diseases. The x‐axis categorizes the specific life stage during which participants were exposed to the famine (ranging from in utero to 31–40 years of age). The y‐axis displays the adjusted Odds Ratios (aOR) for each chronic disease. The horizontal dashed grey line denotes the null value (aOR = 1.0). Estimates positioned above the dashed grey line (i.e., green data points) signify higher odds of disease development (aOR > 1.0), whereas estimates positioned below the dashed grey line (i.e., orange data points) signify lower odds (aOR < 1.0) relative to the unexposed cohort. Confidence intervals that do not intersect the null line indicate statistically significant associations at the *p* < 0.05 level. Asterisks indicate the level of statistical significance: *p < 0.05, **p < 0.01, and ***p < 0.001. Models were adjusted for age, sex, later‐life residence, current marital status, working status, family financial status in childhood, educational attainment, household income, number of diseases in childhood, and number of diseases in adulthood.
**Figure S4:** Three‐way interaction of famine exposure, life stage, and sex on the risk of 14 later‐life non‐communicable chronic diseases. The x‐axis categorizes the specific life stage during which participants were exposed to the famine (ranging from in utero to 24–40 years of age). The y‐axis displays the adjusted Odds Ratios (aOR) for each chronic disease. Red data points represent females and blue data points represent males. The horizontal dashed grey line denotes the null value (aOR = 1.0). Estimates positioned above the dashed grey line signify higher odds of disease development (aOR > 1.0), whereas estimates positioned below the dashed grey line signify lower odds (aOR < 1.0) relative to the unexposed cohort. Confidence intervals that do not intersect the null line indicate statistically significant associations at the *p* < 0.05 level. Asterisks indicate the level of statistical significance: *p < 0.05, **p < 0.01, and ***p < 0.001. Models were adjusted for age, later‐life residence, current marital status, working status, family financial status in childhood, educational attainment, household income, number of diseases in childhood, and number of diseases in adulthood.
**Table S1:** Mean age of onset for 14 chronic diseases, stratified by famine exposure status.
**Table S2:** Multicollinearity diagnostics for the sensitivity models adjusting for lifestyle variables and socioeconomic status.

## Data Availability

The data that support the findings of this study are available in CHARLS data at https://charls.charlsdata.com/pages/data/111/en.html. These data were derived from the following resources available in the public domain.
