# Supplementary material for: Famine Exposure Has Disease‐Specific, Age‐Specific and Sex‐Specific Effects on Different Non‐Communicable Diseases in Later Life: Longitudinal Evidence From China
Source: Matern Child Nutr. 2026 Oct 27;22(4):e70276. doi: 10.1111/mcn.70276 (PMC13714563; doi:10.1111/mcn.70276)
Supplement: Supplementary file 1 [file MCN-22-e70276-s001.docx]

**Supplementary Appendix**

**Table S1. Mean age of onset for 14 chronic diseases, stratified by famine exposure status.**

| Chronic disease | Overall  (*N* = 11,047) | Exposed  (*N* = 9,221) | Unexposed  (*N* = 1,826) | Mean Difference | *P*-value |
| --- | --- | --- | --- | --- | --- |
|  | Mean (SD) | Mean (SD) | Mean (SD) |  |  |
| Hypertension | 60.22 (10.58) | 60.21 (10.44) | 60.23 (11.23) | -0.02 | 0.979 |
| Dyslipidemia | 60.70 (9.56) | 60.63 (9.37) | 61.10 (10.55) | -0.47 | 0.340 |
| Diabetes | 61.78 (8.82) | 61.63 (8.72) | 62.59 (9.34) | -0.96 | 0.086 |
| **Heart disease** | **59.63 (11.54)** | **59.44 (11.37)** | **60.59 (12.38)** | **-1.15** | **0.049** |
| Stroke | 61.97 (11.55) | 61.96 (11.37) | 62.05 (12.54) | -0.09 | 0.823 |
| Lung disease | 56.80 (13.94) | 56.62 (13.84) | 57.78 (14.42) | -1.16 | 0.132 |
| **Asthma** | **57.55 (13.75)** | **56.91 (13.48)** | **60.84 (14.69)** | **-3.93** | **0.001** |
| Liver disease | 55.82 (13.82) | 55.65 (13.84) | 56.78 (13.73) | -1.13 | 0.336 |
| Digestive disease | 52.44 (14.45) | 52.32 (14.39) | 53.17 (14.84) | -0.85 | 0.199 |
| **Psychiatric disease** | **55.54 (14.60)** | **54.82 (14.99)** | **59.77 (11.29)** | **-4.95** | **0.004** |
| **Memory-related disease** | **65.84 (10.59)** | **65.49 (10.40)** | **67.67 (11.39)** | **-2.18** | **0.035** |
| Cancer | 60.23 (11.72) | 60.06 (11.61) | 61.11 (12.34) | -1.05 | 0.548 |
| **Kidney disease** | **58.11 (12.69)** | **57.80 (12.53)** | **60.07 (13.54)** | **-2.27** | **0.014** |
| **Arthritis** | **52.33 (13.16)** | **52.19 (13.10)** | **53.18 (13.47)** | **-0.99** | **0.047** |

Notes: Data are presented as mean (standard deviation). Mean differences represent the difference in the age of onset between the exposed and unexposed groups. Bold font indicates statistically significant differences (*p* < 0.05) between the cohorts.

**Table S2. Multicollinearity diagnostics for the sensitivity models adjusting for lifestyle variables and socioeconomic status.**

| Covariate | Df | Adjusted GVIF |
| --- | --- | --- |
| Famine exposure | 1 | 1.02–1.03 |
| Age | 1 | 2.38–2.60 |
| Life stage at famine exposure | 6 | 1.16–1.18 |
| Sex | 1 | 1.47–1.54 |
| Later-life residence | 1 | 1.10–1.13 |
| Current marital status | 1 | 1.07–1.10 |
| Family financial status in childhood | 1 | 1.03–1.04 |
| Educational attainment | 2 | 1.03–1.05 |
| Main occupation | 1 | 1.18–1.21 |
| Household income | 1 | 1.09–1.14 |
| Number of diseases in childhood | 1 | 1.01–1.02 |
| Number of diseases in adulthood | 1 | 1.02–1.03 |
| Physical exercise | 1 | 1.09–1.13 |
| Drinking | 1 | 1.15–1.17 |
| Smoking | 1 | 1.37–1.44 |

Notes: Adjusted GVIF denotes the generalised variance inflation factor scaled by degrees of freedom, GVIF^(1/(2*Df)) (Fox & Monette, 1992). A value of 2.24 is equivalent to a conventional VIF of 5. Values are the range across the 14 disease-specific models.

**Figure S1. Disease-specific associations between famine exposure (at any age) and the risk of 14 later-life non-communicable diseases.** Participants were classified as exposed only if they self-reported exposure and their residence during famine years matched to one of the 28 provinces documented to have experienced excess mortality. Black squares denote the point estimates for the adjusted Odds Ratios (aOR), and the horizontal lines represent the corresponding 95% Confidence Intervals (CI). The vertical red line indicates the null value (aOR = 1.00). Estimates positioned to the right of the red line signify an increased risk associated with the exposure. Confidence intervals that do not intersect the null line indicate statistically significant associations at the *p* < 0.05 level. Models were adjusted for age, sex, later-life residence, current marital status, working status, family financial status in childhood, educational attainment, household income, number of diseases in childhood, and number of diseases in adulthood.

**Figure S2. Disease-specific associations between famine exposure (at any age) and the risk of 14 later-life non-communicable diseases.** Black squares denote the point estimates for the adjusted Odds Ratios (aOR), and the horizontal lines represent the corresponding 95% Confidence Intervals (CI). The vertical red line indicates the null value (aOR = 1.00). Estimates positioned to the right of the red line signify an increased risk associated with the exposure. Confidence intervals that do not intersect the null line indicate statistically significant associations at the *p* < 0.05 level. Models were adjusted for age, sex, later-life residence, current marital status, working status, family financial status in childhood, educational attainment, household income, occupation, number of diseases in childhood, number of diseases in adulthood, and lifestyle (smoking, drinking, and physical exercise).

**Figure S3.** **Age-specific associations between life stage at famine exposure (eight groups) and the risk of 14 later-life non-communicable diseases.** The x-axis categorizes the specific life stage during which participants were exposed to the famine (ranging from in utero to 31–40 years of age). The y-axis displays the adjusted Odds Ratios (aOR) for each chronic disease. The horizontal dashed grey line denotes the null value (aOR = 1.0). Estimates positioned above the dashed grey line (i.e., green data points) signify higher odds of disease development (aOR > 1.0), whereas estimates positioned below the dashed grey line (i.e., orange data points) signify lower odds (aOR < 1.0) relative to the unexposed cohort. Confidence intervals that do not intersect the null line indicate statistically significant associations at the *p* < 0.05 level. Asterisks indicate the level of statistical significance: * *p* < 0.05, ** *p* < 0.01, and *** *p* < 0.001. Models were adjusted for age, sex, later-life residence, current marital status, working status, family financial status in childhood, educational attainment, household income, number of diseases in childhood, and number of diseases in adulthood.

**Figure S4. Three-way interaction of famine exposure, life stage, and sex on the risk of 14 later-life non-communicable chronic diseases.** The x-axis categorizes the specific life stage during which participants were exposed to the famine (ranging from in utero to 24–40 years of age). The y-axis displays the adjusted Odds Ratios (aOR) for each chronic disease. Red data points represent females and blue data points represent males. The horizontal dashed grey line denotes the null value (aOR = 1.0). Estimates positioned above the dashed grey line signify higher odds of disease development (aOR > 1.0), whereas estimates positioned below the dashed grey line signify lower odds (aOR < 1.0) relative to the unexposed cohort. Confidence intervals that do not intersect the null line indicate statistically significant associations at the *p* < 0.05 level. Asterisks indicate the level of statistical significance: * *p* < 0.05, ** *p* < 0.01, and *** *p* < 0.001. Models were adjusted for age, later-life residence, current marital status, working status, family financial status in childhood, educational attainment, household income, number of diseases in childhood, and number of diseases in adulthood.
